# Hierarchical Characterization and Nanomechanical Assessment of Biomimetic Scaffolds Mimicking Lamellar Bone via Atomic Force Microscopy Cantilever-Based Nanoindentation

**DOI:** 10.3390/ma11071257

**Published:** 2018-07-22

**Authors:** Brian Wingender, Yongliang Ni, Yifan Zhang, Curtis Taylor, Laurie Gower

**Affiliations:** 1 Department of Biomedical Engineering, University of Connecticut Health Center, Farmington, CT 06030-165, USA; wingender@uchc.edu; 2 Department of Mechanical and Aerospace Engineering, University of Florida, Gainesville, FL 32611, USA; niutouchaoren@ufl.edu (Y.N.); yifanzhang97@ufl.edu (Y.Z.); curtis.taylor@ufl.edu (C.T.); 3 Department of Materials Science and Engineering, University of Florida, Gainesville, FL 32611, USA

**Keywords:** cholesteric collagen, bone apatite, cantilever-based AFM nanoindentation, biomimetic mineralization, PILP, liquid crystalline collagen, cholesteric, lamellar, intrafibrillar mineral

## Abstract

The hierarchical structure of bone and intrinsic material properties of its two primary constituents, carbonated apatite and fibrillar collagen, when being synergistically organized into an interpenetrating hard-soft composite, contribute to its excellent mechanical properties. Lamellar bone is the predominant structural motif in mammalian hard tissues; therefore, we believe the fabrication of a collagen/apatite composite with a hierarchical structure that emulates bone, consisting of a dense lamellar microstructure and a mineralized collagen fibril nanostructure, is an important first step toward the goal of regenerative bone tissue engineering. In this work, we exploit the liquid crystalline properties of collagen to fabricate dense matrices that assemble with cholesteric organization. The matrices were crosslinked via carbodiimide chemistry to improve mechanical properties, and are subsequently mineralized via the polymer-induced liquid-precursor (PILP) process to promote intrafibrillar mineralization. Neither the crosslinking procedure nor the mineralization affected the cholesteric collagen microstructures; notably, there was a positive trend toward higher stiffness with increasing crosslink density when measured by cantilever-based atomic force microscopy (AFM) nanoindentation. In the dry state, the average moduli of moderately (X51; 4.8 ± 4.3 GPa) and highly (X76; 7.8 ± 6.7 GPa) crosslinked PILP-mineralized liquid crystalline collagen (LCC) scaffolds were higher than the average modulus of bovine bone (5.5 ± 5.6 GPa).

## 1. Introduction

Mineralized collagen fibrils are the primary building blocks of nearly all types of mammalian hard tissue. At the nanoscale, rope-like collagen fibrils are reinforced by intercalated apatite mineral nanocrystals to form a composite structure that is stiff and strong, yet also very tough. At the microscale, collagen fibrils are arranged in various tissue-specific patterns, which are presumably adapted for a variety of specific mechanical functions [1]. The predominant microscale motif of bone is a lamellar organization in which the orientation of parallel fibril arrays is different in successive layers creating a complex “rotated plywood-like structure” [2]; such lamellar microstructures of bone and other natural materials have been thoroughly reviewed in the literature [3,4,5]. Moreover, lamellar bone lies at the foundation of larger microscale features, such as osteons and trabeculae, which comprise the bulk of compact and spongy bone types, respectively. Thus, the intrinsic material properties of soft collagen fibrils and rigid apatite crystals, and importantly, the hierarchical arrangements in which they are organized, lead to the remarkable load-bearing mechanical properties of compact bone. Despite decades of research into the development of synthetic bone analog materials, autologous bone graft remains as the current gold standard for the repair of a critical-sized defect; however, due to limited supply and donor site morbidity, there is considerable interest in the development of a synthetic alternative.

There are a variety commercial options currently available, but none of these meet the requirements for the regeneration of damaged, load-bearing bone, and most have serious disadvantages over donor bone [6]. The burgeoning field of bone regenerative engineering aims to replace old or damaged tissue with a material that elicits the patients natural repair mechanisms, with the goal of replacing a temporary synthetic matrix by the regeneration of healthy tissue [7]. Several approaches using various material systems are currently being used to generate composite scaffolds with multi-scale hierarchical ordering, however none of them meet the demanding requirements for bone tissue regeneration [8]. The synthetic scaffold should resemble the hierarchical structure of bone in order to provide an extracellular microenvironment that is able to support and stimulate cell-mediated tissue regeneration, serving as a supportive platform for transplanted cells, or recruiting and retaining endogenous cells by providing appropriate mechanical cues and biological triggers [9]. At a minimum, scaffolds should be biocompatible, biodegradable, and/or bioresorbable, osteoinductive, and osteoconductive, support the attachment, proliferation, and migration of cells, and have compatible mechanical properties with the patient’s tissue [10]. Baskin et al. recently developed a novel dense nanophase bone substitute for craniofacial load-bearing applications and showed that highly porous materials may not be necessary for successful cell infiltration and integration with surrounding subcutaneous tissue [11]. Furthermore, they demonstrated that a decrease in porosity did not impact biological response in a rat mandibular defect model; however, a glycation mediated crosslinking strategy with the goal of improving mechanical properties appeared to prevent osteointegration of the implant [12].

Recent attempts by our group to achieve in vitro intrafibrillar mineralization of dense collagen substrates by first demineralizing native bone specimens and subsequently remineralizing them via the polymer-induced liquid-precursor (PILP) process have revealed that the mineral phase did not fully infiltrate the scaffold interior, with a depth of penetration limited to roughly 100 microns from the sample surface [13,14]. Other groups have used compression techniques to expel water and condense polymerized collagen hydrogels; though despite achieving moderately high collagen density, these disorganized networks of collagen fibrils lack the dense parallel-fibered rotating plywood microstructure of lamellar bone [15,16]. Nevertheless, Li and coworker’s plastic compression technique generated dense reconstituted collagen scaffolds which upon PILP mineralization resulted in a mineralized collagen fibril nanostructure and a disorganized microstructure with mechanical properties that are similar to woven bone [17]. Furthermore, they used carbodiimide chemistry to generate various collagen crosslink densities, which appeared to promote both enhanced mineralization and more favorable mechanical properties, citing up to 64 wt % mineral and a conventional average nanoindentation modulus slightly below 10 GPa for the X82 (82% crosslink density) experimental group [17]. Crosslinking of collagen using carbodiimide chemistry (i.e., *N*,*N*-(3-dimethylaminopropyl)-*N*′-ethyl-carbodiimide hydrochloride (EDC) in combination with N-hydroxysuccinimide (NHS)) resulted in a more complete reaction, as compared to EDC alone. This method leads to the formation of amide linkages between collagen molecules, and all of the excess reagents can be removed [18].

Traditional mechanical test configurations are unable to accommodate the small size of microscale bone components, such as an individual osteon or trabeculae (~200 microns in thickness); therefore, Berkovich-style nanoindentation has quickly been adopted as a standard technique to assess the mechanical properties of bone at smaller length scales [19]. Several groups have designed experiments that sought to measure mechanical properties at or below the level of individual collagen fibrils using cantilever-based atomic force microscopy (c-bAFM) nanoindentation [20,21,22,23,24,25]. Androitis et al. proposed a standardization of methodology for carrying out experiments and analyzing data with respect to collagen fibrils and other nanoscale tissue components and material systems [26]. Katsamenis et al. used μ-Raman microscopy in conjunction with c-bAFM nanoindentation on bone to show a correlation between compositional differences in non-collagen protein (NCP) content between adjacent lamellae, which they suggest can lead to selective stiffening under load [27]. Balooch et al. used c-bAFM nanoindentation to show a decrease in modulus of individual dentin collagen fibrils from 1.5 GPa to 50 MPa during acidic demineralization treatment [28]. Based on these successful experiments, c-bAFM nanoindentation was chosen here to investigate how crosslinking treatments effect liquid crystalline collagen (LCC) matrix stiffness at the nanoscale, before and after mineralization.

Previously, we have shown that dense LCC scaffolds with cholesteric microstructures serve as a template for organizing oriented intrafibrillar apatite nanocrystals that are produced during PILP mineralization into a higher level of organization, and this resulted in a hierarchical composite material with bone-like nano- and micro-structures [29]. Here, we report on similar biomimetic lamellar bone-like scaffolds, but now examine varying degrees of crosslink density as a means for tailoring the composite stiffness. We aim to fabricate a collagen-apatite composite material, with tailorable stiffness and compact bone-like hierarchical organization, which would satisfy several requirements as a potentially resorbable, biomimetic scaffold for use in bone regenerative engineering. There were two main goals of this study: (1) to determine whether liquid-crystalline collagen substrates can be fabricated with various crosslink densities using carbodiimide chemistry, and whether or not the crosslinking would influence their ability to mineralize via the PILP process; and, (2) to evaluate the effect of crosslinking on the collagen-apatite composite’s nanoscale mechanical properties. Thus, densely packed, cholesteric collagen films were crosslinked to various densities using carbodiimide chemistry, followed by biomimetic mineralization via the PILP process. The crystal phase and the cholesteric morphologies of the LCC films were characterized by polarized light microscopy (PLM), scanning electron microscopy (SEM), transmission electron microscopy (TEM) with selected area electron diffraction (SAED), atomic force microscopy (AFM), Raman spectroscopy, thermogravimetric analysis (TGA), and wide angle X-ray diffraction (WAXD). The nanoscale mechanical properties were tested by measuring indentation moduli of the mineralized scaffolds using cantilever-based AFM nanoindentation (c-bAFM).

## 2. Materials and Methods

### 2.1. Liquid Crystal Collagen Preparation

Type-I collagen molecules (e.g., tropocollagens) were acid-extracted from Wistar rat tail tendon and purified via selective salt precipitation using an established protocol [30]. Acidic type-I collagen solution was prepared by solubilizing 30 mg of collagen in pre-chilled 0.5 M acetic acid and passing the solution through a syringe filter with 0.22 micron pore size. A final collagen concentration of 3 mg/mL was obtained by adding 1 mL of pre-chilled 10X PBS to the 9 mL of filtered collagen solution, followed by gentle mixing with the pipette to prevent bubble formation. LCC substrates were prepared by injecting the collagen solution inside Slide-a-Lyzer dialysis cassettes (Fisher Scientific, Hampton, NH, USA, MWCO 3.5 kDa), and by performing reverse dialysis for one week at 4 °C against an acidic polyethylene glycol (PEG) solution (40% *w/v* in 1X PBS and 0.5 M acetic acid, pH ~ 3) to concentrate the collagen molecules. The LCC was neutralized by switching the dialysis cassette to a neutralized PEG solution (40% *w/v* in 1X PBS, pH ~ 7.4) at 25 °C, which was then warmed to 37 °C and incubated for one week. After incubation, the LCCs were removed from their dialysis cassettes and washed in 1X PBS, three times for 20 min each, to remove any residual PEG [29].

### 2.2. Carbodiimide Crosslinking

The LCC cross-linking was conducted in a solution of 50 mM 2-(N-morpholino) ethanesulfonic acid (MES) hydrate (pH adjusted to 7.0 with 100 mM NaOH) with increasing concentrations (10, 50, or 100 mM) of 1-ethyl-3-(3-dimethylaminopropyl)carbodiimide hydrochloride (EDC) and *N*-hydroxysulfosuccinimide (NHS) at a constant molar ratio of 2:1 overnight. EDC concentrations were calculated based on constant molar ratios of EDC/NHS and EDC/NH_2_collagen (where NH_2_collagen is an estimate of lysine content) under the assumption that the molecular weight of collagen is 300,000 g/mol with a lysine content of 25/1000 residues/α-chain [31]. The reaction was quenched by immersing collagen films in a solution (0.1 M Na_2_HPO_4_ and 2 M NaCl) for 2 h to hydrolyze any remaining activated carboxyl groups or EDC groups. A non-crosslinked control scaffold underwent the same experimental procedures without the addition of EDC/NHS to the MES buffer. LCC films were rinsed in 50 mM MES, three times for 5 min each, with small specimens that were reserved for TEM analyses, before a final rinse in DI water to remove residual salts prior to mineralization.

### 2.3. Crosslink Density Determination

UV-Vis absorbance was measured to approximate the crosslinking density of each experimental group based on the free amine groups remaining on the collagen [32]. Briefly, collagen samples (~4 mg) were placed in a solution containing 1 mL of 2,4,6-trinitrobenzene sulfonic acid (TNBS, aqueous 0.5% *w*/*v*) and 2 mL of NaHCO_3_ buffer (4% *w*/*v*) and heated for 2 h at 40 °C. Samples were then hydrolyzed by adding 3 mL of HCl (6 N) and heating to 60 °C for 1.5 h. After cooling to room temperature, the solution was diluted to 60 mL. A blank was created in the same manner without collagen. The absorption peak near 345 nm upon the reaction of TNBS with ε-amino groups of L-lysine was recorded using a UV−vis spectrophotometer (Perkin-Elmer Lambda 800, Waltham, MA, USA). The crosslinking densities were calculated from the reduction of absorbance per weight of collagen when compared to non-crosslinked collagen.

### 2.4. PILP Mineralization

Equal volumes of 9 mM CaCl_2_·2H_2_O (Sigma, St. Louis, MO, USA) and 4.2 mM K_2_HPO_4_ (Sigma, St. Louis, MO, USA) solutions were prepared in Tris-buffered saline (TBS) containing 0.9% (*w*/*v*) NaCl and 0.02% (*w*/*v*) sodium azide (Sigma, St. Louis, MO, USA) to prevent microbial activity. The PILP process-directing agent, OPN derived from bovine milk (Lacprodan OPN-10, donated by Arla Foods Ingredients Group P/S, Aarhus, Denmark), was added at a concentration of 100 μg/mL to 200 mL of calcium solution before mixing with an equal volume of the phosphate solution. The LCC scaffolds were incubated in the mineralization solution at 37 °C to emulate the physiological conditions under gentle stirring (stir bar 150 rpm) for 72 h. The mineralized samples were removed from the solution, washed with DI water (3× for 15 min), lyophilized, and stored at 20 °C until further use or embedded in resin for further characterization.

### 2.5. Resin Embedding and Sectioning for Electron and Atomic Force Microscopy

Non-mineralized and mineralized LCC specimens were cut from the bulk, dehydrated, and embedded in acrylic resin, according to the manufacturer’s specifications with slight modifications. Briefly, samples were left unfixed (to preserve the experimental crosslink densities) and were dehydrated through a series of graded ethanol (50% for 15 min, 70% for 15 min, 90% for 15 min, 100% three times for 10 min each) prior to resin embedding and thermal curing (LR White Hard, Electron Microscopy Sciences, Hatfield, PA, USA). For TEM analyses, ultrathin sections (~90 nm) were cut with a diamond knife on a Leica Ultracut T at a rate of 8 mm/s and floated on double-distilled water. Sections were collected on an amorphous carbon-coated 200 mesh nickel type 5a TEM grids (Ted Pella, Inc., Redding, CA, USA). Some non-mineralized LCC samples were stained with heavy metal salts (8% uranyl acetate aq.) to enhance contrast. Immediately after collecting the ultra-thin sections for each group, the microtome settings were changed to cut semi-thin sections (~1.5 μm) for AFM that would correspond to the TEM sections. Semi-thin sections were adsorbed onto a glass slide for subsequent AFM imaging and mechanical testing.

### 2.6. Light and Electron and Atomic Force Microscopy

Semi-thin sections were observed with an Olympus BX60 microscope (Tokyo, Japan) between crossed-polarizers. Molecular orientations were visualized with a gypsum first-order red retardation plate inserted at 45° between the polarizer and analyzer [33]. Images were captured with a Lumenera Infinity 3 digital camera (Ottawa, ON, Canada). Freeze-dried samples were mounted onto aluminum stubs via adherence to double-sided copper tape. All of the samples were then sputter coated with amorphous carbon before analysis with an FEI Nova 430, operated at 15 kV and a spot size of 4.

Ultrathin sections were imaged with a Hitachi 760 TEM (Hitachi High-Technologies America, Schaumburg, IL, USA) equipped with a Macrofire monochrome progressive scan CCD camera (Optronics, Goleta, CA, USA) and AMT image capture software version 600.335p (Advanced Microscopy Techniques, Danvers, MA, USA) at an accelerating voltage of 80 kV in bright field TEM (BF-TEM) mode. To help to visualize the crystallographic orientation of the hydroxyapatite crystals, ultrathin sections were imaged in BF/SAED mode using a JEOL 2010F (Tokyo, Japan) operating at 200 kV. AFM imaging was conducted on an Asylum MFP-3D (Santa Barbara, CA, USA) operating in AC tapping mode with a nominal scan rate of 1 Hz.

### 2.7. Wide Angle X-Ray Diffraction

Mineralized samples were scanned with Cu Kα X-ray radiation from a Philips XRD ADP 3720 diffractometer (Amsterdam, The Netherlands) at 40 kV and 45 mA, using a step size of 0.01° mrad·s^−1^ with a time of 10 sec/step, over a 2θ range of 10–60°.

### 2.8. Thermogravimetric Analysis

A heating rate of 20 °C·min^−1^ was applied to mineralized samples in the temperature range 30–800 °C under nitrogen at a flow rate of 100 mL·min^−1^ using a TG/DTA 320 (Seiko, Thermo Haake, Karlsruhe, Germany) instrument.

### 2.9. Raman Spectroscopy

Micro-Raman spectroscopy was performed using a Renishaw inVia system (Wharton Anderch, UK) with a wavelength of 633 nm and a spot size of ~5 μm projected through a 50× objective with numerical aperture equal to 0.5 and an excitation power of 5 mW.

### 2.10. Cantilever-Based AFM Nanoindentation

AFM experiments were performed in both dry and wet ambient conditions at room temperature. Cantilever-based nanoindentation was conducted in contact mode using PPP-NCHR (Nanosensors, <10 nm tip radius and ~40 N/m spring constant) cantilevers. AFM AC mode (or tapping) images were acquired prior to indentation, and these 40 × 40 micron areas were used to identify Regions of Interest (ROI) having lamellar features. After imaging a 1 × 1 micron ROI, 100 force-controlled indentations were performed in two separate ROIs per experimental group. The samples were equilibrated in the closed AFM chamber for at least 30 min before testing. A second set of samples was prepared to perform indentation tests in aqueous conditions by gluing a small piece of “bulk” LCC to a glass slide using Crystalbond™ 509 adhesive (Electron Microscopy Sciences, Hatfield, PA, USA), such that a flat part of the sample surface was exposed. Indentation experiments with similar parameters were performed for mineralized samples in both dry and hydrated states, the samples were immersed in 1X PBS and allowed to equilibrate for at least 4 h in a liquid cell (CCELL, Asylum Research, Santa Barbara, CA, USA) prior to indentation in the hydrated state. Because of the different testing conditions, in the submerged and dry states, the calibration of optical lever sensitivity was performed as described below for both configurations.

Inverse optical lever sensitivity (InvOLS) and cantilever spring constant calibration were performed using the manufacturer’s Get Real™ function (Asylum Research, Inc., Santa Barbara, CA, USA), which is based on the Sader method [34]. Calibration of error in air of the optical path (virtual deflection) was achieved by performing full range of z-piezo ramping in air and submerged conditions. Cantilever InvOLS was determined by averaging 50 measured slopes of the indentation curves against an “infinite” hard surface (single crystal sapphire). The spring constant was determined to be ~34 N/m, approximately the value given by the manufacturer (37 N/m). An estimation of the tip radius was determined from contact mode scanning across the NioProbe surface (Aurora NanoDevice Inc., Nanaimo, BC, Canada). The reduced modulus (E_R_) was determined by direct fitting the force-indentation curve with a proper contact model. For indentation on dry “bulk” samples, the upper portion (45–95%) of the unloading curves were fitted by the Oliver-Pharr model while assuming spherical on flat contact geometry [35]. For submerged indentation, Hertzian contact model was applied to fit the loading curve under the assumption of purely elastic and non-adhesive contact [36,37].

### 2.11. Statistical Analysis

Welch’s one-way analysis of variance (ANOVA), and the Games-Howell post-hoc method of multiple comparisons, was performed to assess statistically significant differences (*p*-value ≤ 0.01) between groups with unequal variance. Unpaired two-tailed t-tests were used to detect the differences between groups with equal variance (*p*-value < 0.01). Statistical analyses were performed in MINITAB^®^ 18 (State College, PA, USA) or Microsoft Excel 2016 (Redmond, WA, USA) with the Data Analysis plugin. Tukey Box-and-whisker plots were constructed by calculating the first quartile (Q1), third quartile (Q3), mean and median of the data. The interquartile range (IQR) is defined as the distance between the first and third quartile. Upper and lower whiskers were defined as (Q3 + 1.5 × IQR) and (Q1 − 1.5 × IQR), respectively. Data points outside of the whisker bounds were considered to be outliers.

## 3. Results

Dense collagen films with cholesteric microstructures were generated via the molecular crowding technique that was developed by Saeidi et al. [38]. The films were systematically crosslinked to different densities via carbodiimide reactions between carboxylic acids and amine groups of neighboring collagen molecules following an established protocol [18]. Crosslink density as a function of EDC concentration was calculated as a percentage based on the maximum number of free amino acid groups available and is shown in Table 1 [17]. The crosslinking reactions, including the uncrosslinked control, produced groups with crosslink densities of 0%, 32%, 51%, and 76%, which will be referred to henceforth as X0, X32, X51, and X76, respectively.

**Figure 1:**
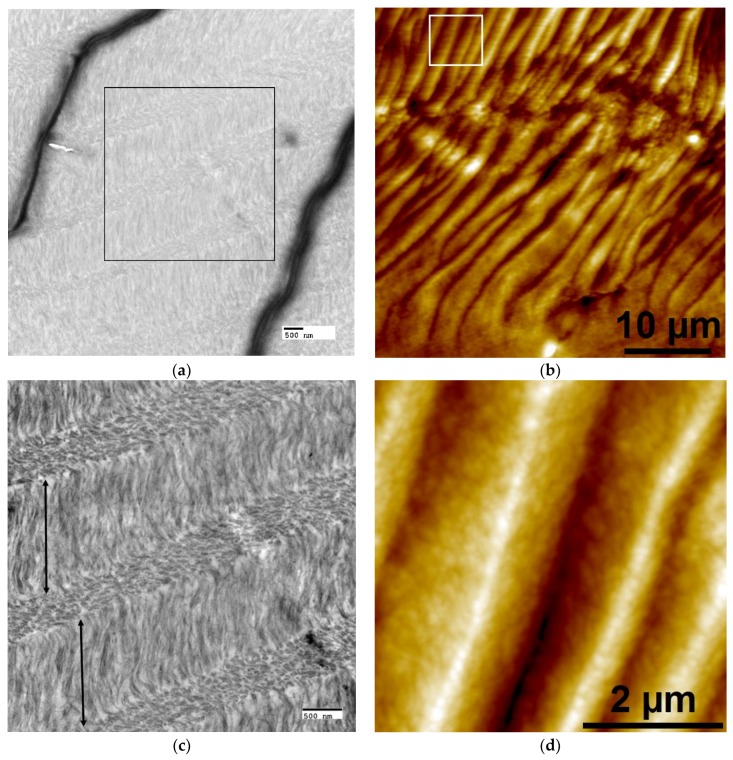

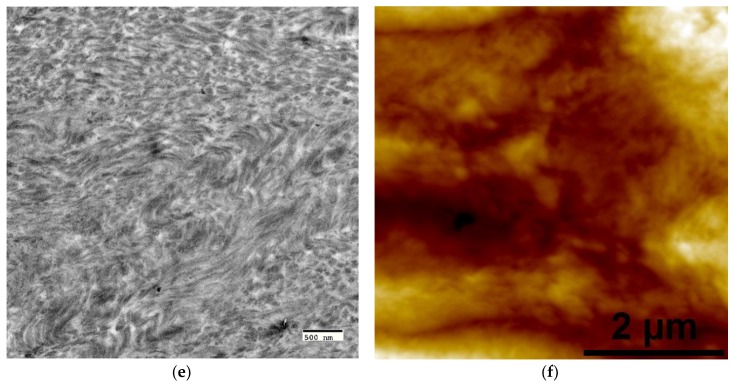
Representative cross-sectional transmission electron microscopy (TEM) (**a**,**c**,**e**) and complementary atomic force microscopy (AFM) height images (**b**,**d**,**f**) showing organized and disorganized regions in crosslinked liquid crystalline collagen (LCC) film X51. (**a**,**b**) Low mag images demonstrate cholesteric collagen microstructural organization due to the changing orientation of parallel-fibril arrays through successive layers. (**a**) Despite uranyl acetate staining, the contrast of non-mineralized LCC was poor at low magnifications due to artifacts (wrinkles, dark lines) created during the ultra-thin sectioning process; the black box denotes the ROI for the image shown in (**c**). (**b**) In AFM, periodic peaks and valleys (an artifact created from interaction of the knife with the twisted plywood structures during semi-thin sectioning) can be visualized as lighter regions corresponding to elevated peaks and darker areas as valleys; the white box denotes the ROI for the image shown in (**d**). (**c**) At higher magnification in TEM, fibrils in lamellae with axes orientated parallel to the image plane appear filamentous (layers denoted by double-sided arrows); in the other layers fibrils oriented perpendicular or oblique to the image plane appear as small ovals. (**d**) At higher magnification in AFM, height images show better contrast from periodic peaks and valleys with sub-micron roughness, likely the result of an oblique orientation of collagen fibrils in successive lamellae [40]. (**e**,**f**) Between large (~10–40 μm) cholesteric domains, small regions (~1–3 μm) of disorganized collagen fibrils could be found. Scale bars: (**a**,**c**,**e**) 500 nm; (**b**) 10 microns; (**d**,**f**) 2 microns.

After the crosslinking reaction, non-mineralized LCC samples were embedded and sectioned for imaging via TEM and AFM to qualitatively examine the collagen organization (Figure 1). Cholesteric microstructures were present in all groups after the crosslinking treatment; X51 was chosen as a representative sample to demonstrate the cholesteric organization of collagen, which is best visualized in TEM when the collagen fibrils are oriented parallel/perpendicular to the image plane. Open arrows delineate dark wrinkles, artifacts of microtome sample preparation that make it difficult to visualize collagen organization at a relatively low magnification, even with staining to enhance contrast (Figure 1a), but the cholesteric ordering is readily seen at higher magnifications (Figure 1c,d). Therefore, complimentary AFM height images were found to be more useful for visualizing cholesteric collagen microstructures over longer distances (Figure 1b and Figure 3b). Lamellar features were present in all of the scaffolds, with layer thicknesses ranging from approximately 0.5 to 5 microns. The lamellar organization in these LCC scaffolds was not homogeneous; thin, disorganized boundaries existed between larger cholesteric domains though they still appeared to be comprised of fibrillar collagen (Figure 1e,f). The pitch and periodicity varied throughout the LCC matrix, presumably a function of collagen concentration, as previously reported [39]. It should be noted that inhomogeneity in cholesteric ordering was likely to be a direct result of geometric restrictions that are imposed by the shape of the dialysis cassette in this experiment. Although outside the primary focus of this paper, Supplementary Figure S1 was prepared to describe, in greater detail, the different collagen structures that were formed in this work. The terminology that was used in Figure S1 was directly adapted from the work of Mosser et al. [39]. Because the focus of the experiment was to develop a material that mimics both the mineralized fibril building block and the lamellar microstructures of bone, cholesteric regions were selectively chosen for further characterization.

**Figure 2:**
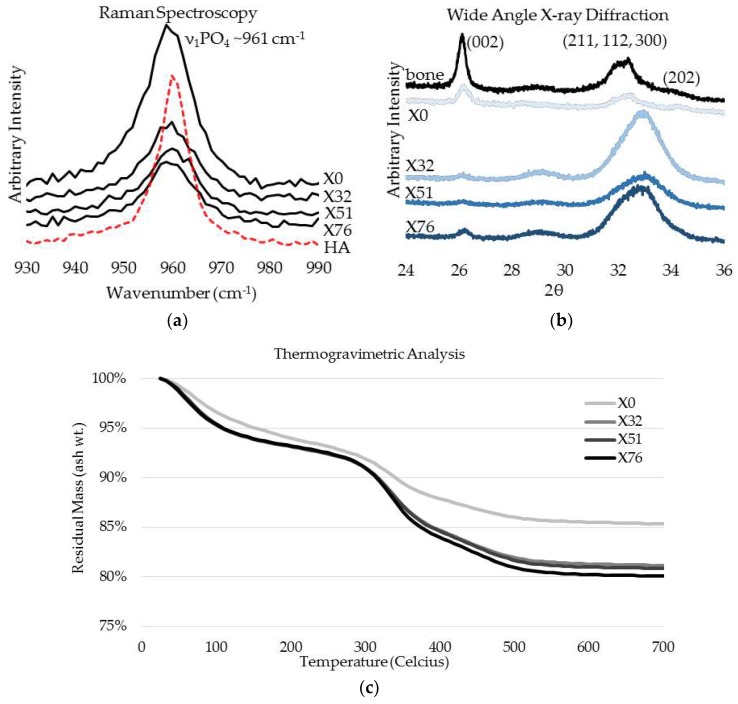
Characterization of crosslinked, polymer-induced liquid-precursor (PILP) mineralized LCC scaffolds. (**a**) Raman spectra from the four experimental groups (black) as compared with that of commercial HA (red dashed line) which has a sharp symmetric peak centered around 961 cm^−1^ due to the ν_1_PO_4_ vibrational mode of crystalline hydroxyapatite; (**b**) WAXS spectra of the four experimental groups compared to a bovine bone; and, (**c**) thermogravimetric analysis (TGA) residual ash weights indicate the scaffolds were highly mineralized and may have contained over 80 wt. % inorganic material.

The OPN-directed PILP mineralization was terminated after 72 h to prevent possible precipitation, which would result in the subsequent formation of an extrafibrillar apatite surface coating. After mineralization, several characterization methods were used to examine the resulting mineral phase, the results of which are summarized in Figure 2. Raman spectroscopy was performed to compare the strongest phosphate vibrational mode (ν_1_PO_4_) of OPN-PILP mineralized collagen to that of commercial hydroxyapatite (Figure 2a). When compared to commercial crystalline hydroxyapatite, which has a sharp ν_1_PO_4_ symmetric peak centered about 961 cm^−1^, PILP-mineralized LCC films had broader, less intense, and less symmetric peaks that appeared to be centered at ~959 cm^−1^. The spectra followed a general trend of decreasing ν_1_PO_4_ Raman intensity with increasing crosslink density. This could be indicative of physical constraints, such as changes to intramolecular spacing due to increased crosslink density, which may restrict elongation and growth of PILP-generated mineral crystals. WAXD spectra were also collected from the OPN-PILP groups and when compared to spectra of native bovine bone the difference in peak ratios suggest that crosslinking may help to preserve the orientation of collagen fibrils (i.e., containing intrafibrillar mineral) near the surface of crosslinked LCC samples (Figure 2b) [41]. The uncrosslinked control scaffold (X0) had a WAXD spectra that most closely resembled mature bone apatite, with the strongest 2θ peak intensity coming from the (002) plane at 29°, followed by the overlapping peaks from the (211), (112), and (300) near 32°, and the (310) peak at 40°. Conversely, the three crosslinked groups had spectra that were similar to less crystalline, or amorphous, apatite mineral, with a broad 2θ peak around 30° to 35°, consistent with the advancement of apatite crystallization in developing rat calvaria [42]. TGA (n = 1) was used to estimate the amount of mineral in each scaffold by measuring the residual ash weight after water loss (~200 °C) and the combustion of organic constituents at ~600 °C. The inorganic ash weight measurements at 600 °C were approximately 85%, 81%, 81%, and 80%, for the experimental groups X0, X32, X51, and X76, respectively (Figure 2c).

**Figure 3:**
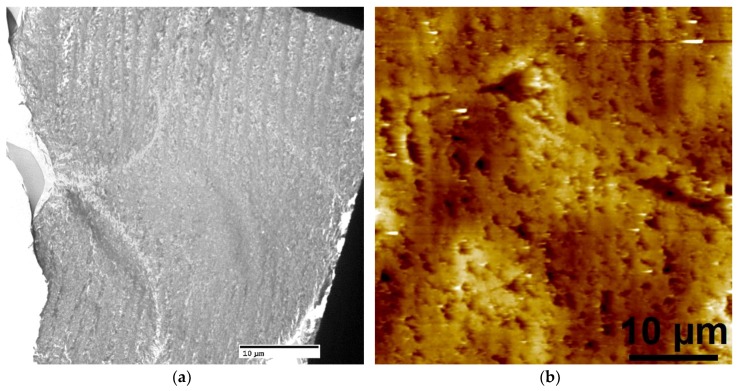
Bright field TEM (BF-TEM) and AFM height image of mineralized LCC cholesteric microstructure and “domain” boundaries. (**a**) The non-crosslinked control, X0, had cholesteric domains that spanned 10’s of microns and appeared to have a globular three-dimensional shape, as judging from circular domains in the two-dimensional image; and, (**b**) Similar features were identified via AFM. Despite being highly mineralized, these structures closely resemble the schematic description of interconnected cholesteric collagen spherulites and isotropic defects that have been observed, and schematically depicted, by Mosser and colleagues [39].

**Figure 4:**
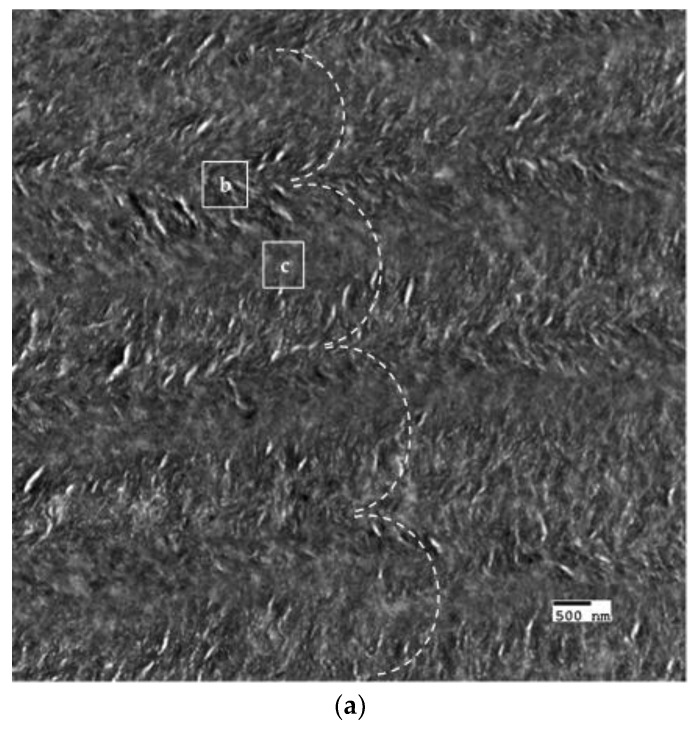

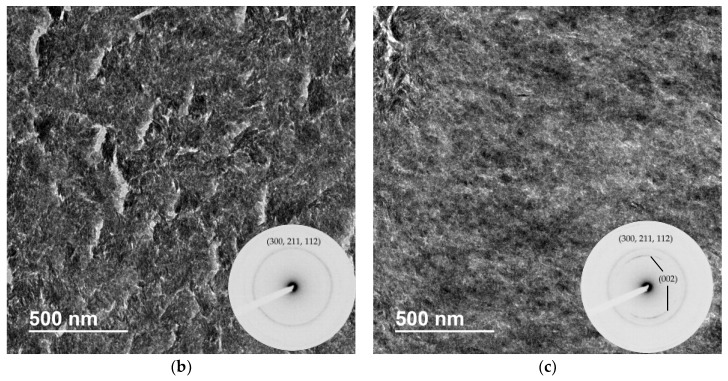
BF-TEM and corresponding selected area electron diffraction (SAED) patterns of PILP mineralized LCC (X32). (**a**) The herringbone pattern results when lamellae are sectioned on a bias; the shifting orientation of collagen fibrils is represented by the series of nested arcs (dashed white line) which create a herringbone pattern. Because the collagen fibrils serve as a template for mineralization, the crystallographic *c*-axes of PILP formed crystals should be oriented along the axis of the collagen fibrils such that SAED can assist in interpreting the image. The regions marked ‘b’ and ‘c’ are shown at a higher magnification with corresponding diffraction patterns in Figure 4b,c, respectively; (**b**) The absence of the (002) reflection in the diffraction pattern (inset) indicates that the zone axis is very close to [001], therefore the fibril axes are oriented perpendicular to the image plane; and, (**c**) The presence of (002) arcs in this diffraction pattern (inset) indicate that the crystallographic *c*-axes are co-oriented with collagen fibril axes and are parallel to the image plane.

**Figure 5:**
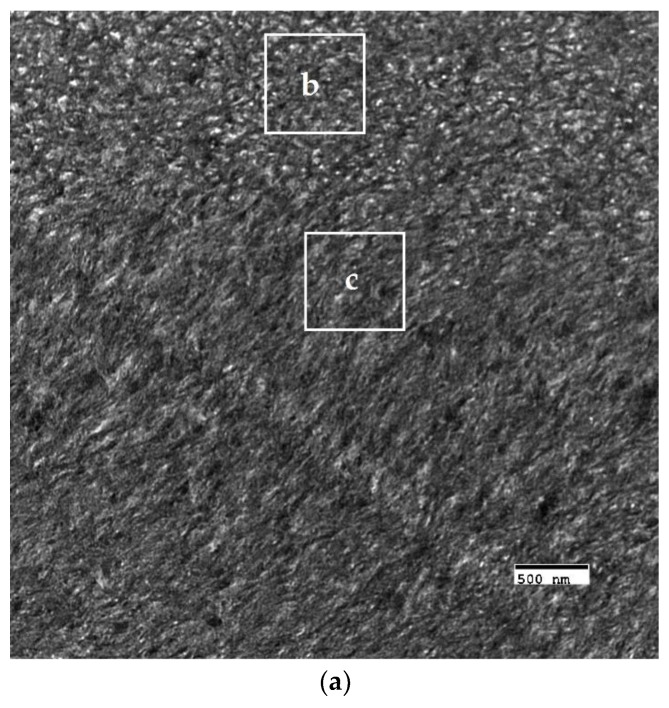

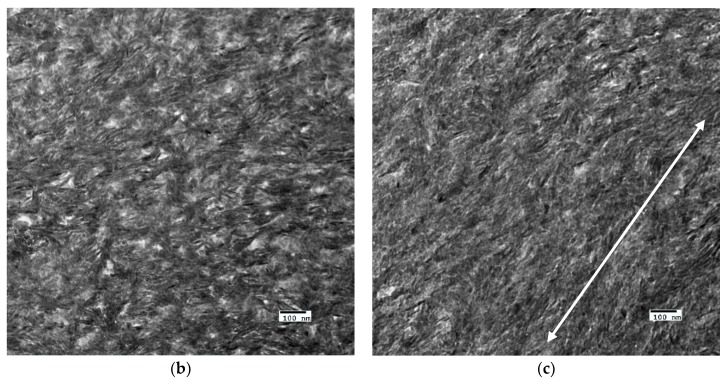
Nanoscale mineral patterns templated by parallel arrays of collagen fibrils in X76, visualized in bright field TEM. (**a**) Low magnification TEM image that shows two different collagen/mineral orientations in neighboring lamellae, the boxes marked ‘b’ and ‘c’ correspond to the high magnification images shown in Figure 5b,c, respectively; (**b**) Acicular mineral pattern similar to the “lacy” regions described by Reznikov et al., whose tomographic reconstructions depicts mineral crystals bending around collagen fibrils when viewed end-on; and, (**c**) Co-alignment of the collagen fibril axes and the fast-growing crystallographic *c*-axes (orientation shown by double-sided white arrow) of elongated mineral crystals results in this “filamentous” pattern, in which the mineral has classically been described as having a needle or plate-like morphology.

The crosslinked scaffolds appeared to be heavily mineralized when viewed in bright field TEM and AFM (Figure 3, Figure 4 and Figure 5). The collagen matrices served as a template for mineralization during the PILP process, the resulting mineral provided adequate contrast to reveal the underlying cholesteric microstructures, which were still present in all groups. Continuous spherical lamellar domains spanned tens of microns with thin, less-organized domain “boundaries” (Figure 3). These regions resemble mineralized versions the “interconnected spherulitic”, “loose cholesteric”, and “dense cholesteric” phases, as characterized by Mosser et al. using a combination of TEM and PLM to visualize dense collagen matrices reconstituted in vitro [39]. The herringbone pattern shown in Figure 4a is a classic cholesteric pattern shown previously in a variety of natural materials and described in exquisite detail by Bouligand [3]. To help interpret the image, and to confirm the intrafibrillar mineralization of collagen fibrils, electron diffraction patterns were obtained from the regions marked ‘b’ and ‘c’ in Figure 4a. The absence of the (002) reflection in the diffraction pattern from region ‘b’ indicates that the crystallographic *c*-axes (e.g., the collagen fibril axes) are oriented nearly perpendicular to the image plane, while the presence of the (002) in the region ‘c’ diffraction pattern shows that the collagen fibrils lie in the plane. Notably, the two regions marked ‘b’ and ‘c’ in Figure 5 correlate nicely with the relatively recently characterized “lacy/nested/acicular” and “filamentous” nanoscale mineral patterns that were observed in compact lamellar bone [43,44].

**Figure 6:**
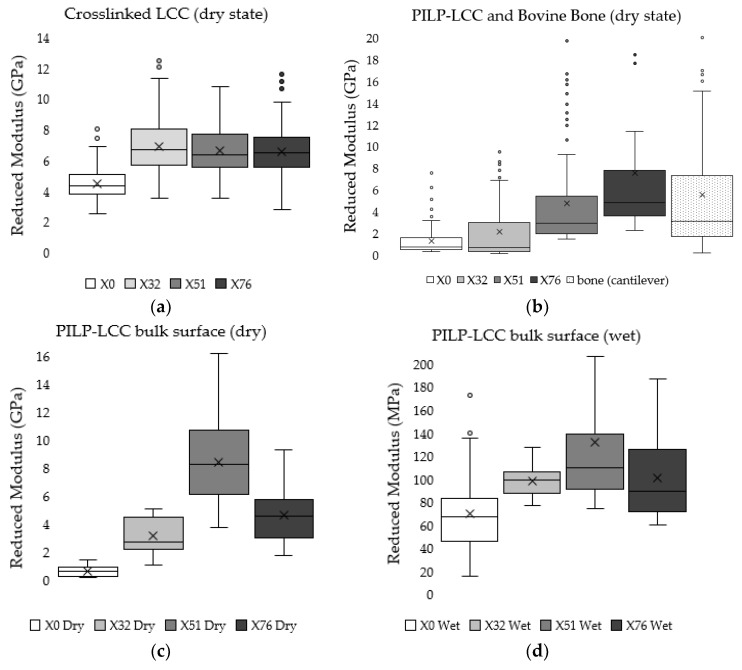
Tukey box-and-whisker plots of indentation moduli help to view the distribution of data from two separate c-bAFM nanoindentation experiments. The box ends represent the upper and lower quartiles, the median is marked by the horizontal line inside the box, the mean is marked by the X, and potential outliers are shown as circles located outside of the whiskers. For a more detailed description of the box-and–whisker plot construction, please see the methods. (**a**) Non-mineralized crosslinked LCC semi-thin cross section tested in the dry state; (**b**) PILP mineralized crosslinked LCC semi-thin cross section compared with native bovine bone; (**c**) Bulk PILP-LCC sample surface measured in the dry state; and, (**d**) Bulk PILP-LCC sample surface measured in liquid after re-hydrating in 1X PBS.

**Figure 7:**
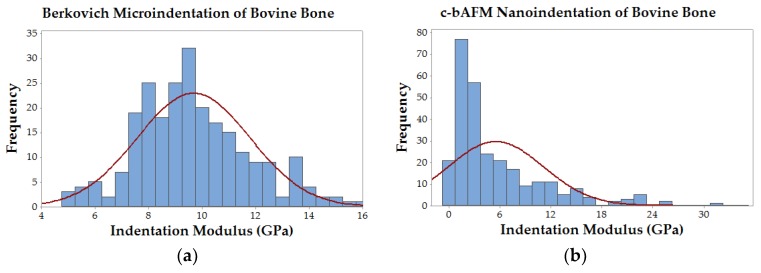
Histograms of modulus data from native bovine bone comparing Berkovich-style microindentation and c-bAFM nanoindentation techniques. (**a**) Berkovich-style microindentation data followed a normal distribution with a mean indentation modulus of 9.7 ± 2.2 GPa; and, (**b**) Cantilever-based AFM nanoindentation data had a right-skewed distribution with a mean indentation modulus of 5.5 ± 5.6 GPa.

Cantilever-based AFM nanoindentation was performed on the crosslinked scaffolds before and after mineralization, and also in the wet and dry states after mineralization, to measure the effect of increasing collagen crosslink density on the material’s nanoscale stiffness. Indentation moduli data are reported graphically as box plots in Figure 6, which helps to visualize the distribution of data, and in the following text as (mean ± standard deviation). It is important to note that moduli that are reported here were obtained at indentation depths of approximately 10 nm. Analysis of force curves collected from non-mineralized LCC scaffolds (Figure 6a) showed no significant differences in the indentation moduli of the three crosslinked groups (*p*-value = 0.05; E_R(X32)_ = 6.9 ± 1.8 GPa, E_R(X51)_ = 6.6 ± 1.5 GPa, E_R(X76)_ = 6.6 ± 1.5 GPa), but all three were significantly stiffer than the non-crosslinked LCC (*p*-value << 0.01; E_R(X0)_ = 4.2 ± 1.0 GPa). In contrast, the moduli of all PILP mineralized groups (Figure 6b) were significantly different from one another, except X0 and X32, which had a *p*-value = 0.019 (*p*-value << 0.01; E_R(X0)_ = 1.3 ± 1.4 GPa, E_R(X32)_ = 2.1 ± 2.7 GPa, E_R(X51)_ = 4.8 ± 4.3 GPa, E_R(X76)_ = 7.8 ± 6.7 GPa). A second set of PILP-LCC samples were mounted in Crystalbond™ and indentation force curves were collected on the surface of “bulk” material in dry ambient conditions, followed by a second set of indentations in the wet state after equilibration in 1X PBS. The indentation modulus values in the dry state (Figure 6c) were all significantly different from one another, but there was not an obvious correlation between crosslink density and modulus (*p*-value < 0.001; E_R(X0dry)_ = 0.6 ± 0.3 GPa, E_R(X32dry)_ = 3.1 ± 1.2 GPa, E_R(X51dry)_ = 8.4 ± 3.3 GPa, E_R(X76dry)_ = 4.6 ± 2.0 GPa). Fully-hydrated (Figure 6d), the moduli of the mineralized scaffolds dropped by approximately an order of magnitude, and again the non-crosslinked LCC was significantly less stiff than the crosslinked groups (*p*-value < 0.01; E_R(X0wet)_ = 69.4 ± 35.4 MPa). Among the crosslinked groups, X51 had the highest average modulus but it was not statistically different from X76 (*p*-value = 0.04; E_R(X51wet)_ = 131.3 ± 57.6 MPa, E_R(X76wet)_ = 100.7 ± 37.7 MPa). Similarly, X32’s modulus was not significantly different from X76 (*p*-value = 0.64), however, X32 was significantly different from X51 (*p*-value < 0.001). Indentation force measurements were made on native bovine bone reference samples with two AFM-based indentation systems (Figure 7); the two techniques produced statistically significant (*p*-value << 0.01) average moduli of 9.7 ± 2.2 GPa and 5.5 ± 5.6 GPa for the Berkovich and cantilever methods, respectively.

## 4. Discussion

Liquid crystalline collagen processing was used to generate dense, fibrillar collagen substrates with cholesteric domains, as based on previous work [38,39,45,46,47]. The scaffolds were subsequently crosslinked using carbodiimide chemistry with increasing concentrations of EDC/NHS, which resulted in four experimental groups with crosslink densities of 0%, 32%, 51%, and 76% [17]. Reverse dialysis using the Slide-a-Lyzer™ cassette did not result in a homogeneous cholesteric collagen scaffold under the current experimental conditions, though the cholesteric regions were easily identifiable with nondestructive imaging techniques, like PLM (see Supplementary Figure S1); therefore, only the cholesteric regions were further characterized. The cholesteric domains in all TEM sections typically persisted for 10–50 microns and had lamellae with thicknesses ranging from approximately 0.5–3 microns. Between cholesteric domains were the “domain boundaries” that appeared to contain disorganized fibrillar collagen with poor contrast in BF-TEM, indicating that they may also be mineral deficient (the author considers these microscale features analogous to nanoscale grain boundaries).

The OPN-directed PILP process resulted in the intrafibrillar mineralization of collagen. OPN was chosen based on its success in a previous experiment that noted more homogeneously mineralized fibrils and favorable in vitro osteoclast activation when compared to a synthetic PILP additive [13]. Despite no apparent differences in crystal size/shape in TEM, the analytical analyses shown in Figure 2 indicate that the crosslinking procedure did affect the PILP mineral, relative to the non-crosslinked group (X0). Because the TGA was conducted in a Nitrogen gas environment (n = 1, due to limited sample size), the ash weight values reported here may be artificially elevated due to the incomplete combustion of organics. However, such high values would not be unheard of, with mineral quantities above 80 wt. % being reported for the highly mineralized ear bones of whales and dolphins [48]. In general, X0 had a higher wt. % mineral content, a higher normalized ν_1_PO_4_ Raman peak intensity, and a WAXD spectra that resembled mature bovine bone. Conversely, the three crosslinked groups had decreasing mineral content, lower Raman peak intensity, and WAXD spectra that were very similar to developing rat calvaria [42]. When compared to the strongest phosphate vibrational mode (ν_1_PO_4_) of commercial hydroxyapatite, which has a sharp symmetric peak centered about 961 cm^−1^, all PILP-mineralized LCC films had much broader, less intense peaks that appeared to either be shifted to a slightly lower wavenumber, or to have a shoulder that may indicate the presence of a secondary, transient phase, such as amorphous calcium phosphate [49]. This is significant because bone mineral is highly substituted, particularly with carbonate impurities that are known to decrease the solubility of hydroxyapatite and may make it easier for cellular resorption during metabolic remodeling [50]. WAXD data reinforced the perception that the minerals were apatitic, nano-sized, and possibly contained a secondary poorly-ordered phase [42]. TEM/SAED data confirmed that elongated intrafibrillar apatite crystals were co-oriented with collagen fibril axes at the nanoscale and further organized at the microscale by the lamellar collagen template [29]. Despite the differences that were indicated by analytical data, there were no qualitative differences between groups in crystal size/shape or nano/micro patterns at the TEM magnifications used in this experiment, though the authors do not rule out the possibility that the mineral phase may have been altered as part of the sample preparation processes (i.e., dehydration).

The indentation moduli were measured via c-bAFM nanoindentation to determine the effect of increasing collagen crosslink density on mineralized composite stiffness. Several recent studies, summarized by Andriotis et al., have reported the stiffness of non-mineralized collagenous tissues and individual fibrils in the dry state to range from 1 to 11.5 GPa, depending on the analysis method and testing conditions, in particular hydration [26]. Additionally, Andriotis et al. present a very useful methodology and analysis approach for AFM cantilever-based nanoindentation experiments [26]. Given our results on the non-mineralized LCC groups, it seems plausible that the crosslink treatment significantly increased the LCC moduli, yet, due to the compacted density and cohesiveness of dry collagen, there were no significant differences in the moduli of X32, X51, and X76 prior to mineralization (Figure 6a). In the dry state, collagen has properties like a plastic; it is only when collagen is swollen with water that it behaves like a hydrogel, where such differences might be observable.

Mineralization drastically changed the LCC mechanical properties, and there was a positive correlation between stiffness and crosslink density (Figure 6b). Surprisingly, there was a reduction in stiffness after mineralization for LCCs without crosslinks (X0) and with some crosslinks (X32), while the moduli for the moderately (X51) and highly crosslinked (X76) scaffolds increased. A second set of AFM experiments were performed to measure the indentation moduli of PILP-mineralized LCC scaffolds in the wet and dry state. There was a drastic reduction in the indentation modulus of fully hydrated samples (Figure 6d) when compared the same samples in the dry state (Figure 6c); however, the groups followed similar trends in both conditions. The X51 sample had an average indentation modulus of about 137 MPa, which is near a value that was reported in the literature for wet woven bone (132 MPa) from a rat fracture callus [17].

When compared to established Berkovich-style microindentation, nano-scale c-bAFM has ultrahigh resolution but it suffers from several sources of error. Two potential error sources in our measurements arise from the limited spring constant of common cantilevers and their sensitivity to contact geometry during indentation. The reliable range of sample moduli for PPP-NCHR cantilevers specified by the manufacturer is capped at roughly 10 GPa, which is close to the predicted value of our highly crosslinked biomimetic bone sample. Andriotis et al. suggest that c-bAFM modulus measurements can be lower and have more variability than conventional nanoindentation, which is consistent with our measurements on native bovine bone reference samples [26]. Some of our experimental group’s moduli (and native bone’s) approached the limit for sample stiffness (e.g., contact stiffness was much higher than cantilever stiffness) which could have created measurement errors due to cantilever deflection. Moreover, the distribution of data from conventional nanoindentation appears to be near normal, while the cantilever data is clearly skewed rightward (Figure 7). The large variation in real area of contact in c-bAFM indentation could originate from the contact depth if it similar in size to the sample surface roughness, which will result in an error in measured modulus. Finally, it is important to consider the difference in scale of contact area between these two techniques; Berkovich-style indentation results in contact depths approaching one micron, while c-bAFM was limited to approximately 10–20 nanometers. For this reason, and others described above, nanoscale inhomogeneity in material stiffness would have a much more pronounced effect on c-bAFM measurements.

Interestingly, to the best of the author’s knowledge, there are only two prior instances of c-bAFM nanoindentation on mammalian hard tissues: Balooch et al. measured the change in modulus of an individual dentin fibril during a demineralization process [15]; and, Katsamenis et al. probed the toughening mechanisms of osteonal bone by measuring the modulus of individual lamellae [14]. Our indentation modulus measurements on native bovine bone using c-bAFM had a significantly lower average value and a larger standard deviation when compared to Berkovich-style indentation data taken from the same samples (Figure 7), which is in agreement with similar data reported by Androitis and coworkers [26]. Notably, our mean modulus value for bovine bone measured by cantilever-based AFM nanoindentation was 5.5 ± 5.6 GPa, which is in between the values that were measured for the mineralized X51 and X72 groups, and slightly higher than the previously reported values for cortical bovine bone lamellae (1.79–2.79 GPa, [27]) and a mineralized collagen fibril from human dentin (1.2–1.5 GPa) [28]. Importantly, these data suggest that carbodiimide crosslinking can be used to systematically alter the stiffness of PILP-mineralized collagen matrices to match, or even exceed, levels that are found in native bone. Moreover, c-bAFM seems like a viable method for evaluating nanoscale material property inhomogenieties, and, as stiffer cantilevers are developed in the future, it should expand the possibility to more accurately measure the properties of extremely hard and stiff materials, such as biominerals.

Recapitulating bone’s complex hierarchical structure and unique combination of mechanical properties has been a goal for materials scientists and engineers for decades. Particularly for the case of a growing pediatric patient, current metallic and ceramic “permanent” implant materials have unacceptable long-term results [51]. A promising strategy for bone regenerative engineering is the development of a three-dimensional material that can either act as a passive scaffold for the attachment, migration, and proliferation of cells; or, as a bioactive material that is capable of inducing cell recruitment and migration from the patient. The collagen-apatite composite described here, with lamellar bone-like organization and mechanical properties could have the potential as such a scaffold if the processing methods can be scaled up. Dense non-mineralized LCC films have recently been shown to support osteoblast proliferation and increase ALP and osteocalcin expression when compared to control scaffolds [52]. Moreover, transparent LCC matrices that were developed to mimic the cornea supported the successful colonization of human corneal epithelial cells, which were able to generate *de novo* a cultured epithelium layer associated with increased matrix transparency [53]. Additionally, a recent review on physiological bone remodeling stressed the need to test novel bone substitute materials in vitro, by mimicking the correct sequence of resorption followed by formation, to assess their potential for bone regenerative engineering applications [54]. We are currently assessing the viability of PILP-mineralized LCC scaffolds for bone regeneration through cellular based remodeling, using scaffold based chemistry and structure to stimulate metabolic remodeling and test it’s potential to form bone.

## 5. Conclusions

In conclusion, we exploited the liquid crystalline properties of collagen to generate fibrillar collagen substrates with cholesteric microstructures. These scaffolds were subjected to a carbodiimide crosslinking treatment, which resulted in four experimental LCCs of varying crosslink densities. The scaffolds were mineralized via an OPN-directed PILP process that led to the controlled deposition of tiny, elongated apatitic minerals that were templated by the collagen fibrils. The hierarchical, lamellar bone-like microstructures and mineralized collagen fibril nanostructure were characterized by electron microscopy and spectroscopic methods. Finally, cantilever-based AFM nanoindentation was performed on the mineralized composites and showed a positive correlation between the increasing crosslink density and nanoscale stiffness. The results of these experiments suggest that carbodiimide crosslinking does not qualitatively change LCC microstructures and it can be used to systematically crosslink collagen to different densities and thus different stiffness. For comparison, cantilever-based AFM nanoindentation was also used to measure the moduli of native bovine bone and PILP-LCC bone mimetics; the average indentation moduli of moderately (X51, 4.8 ± 4.3 GPa) and highly (X76, 7.8 ± 6.7 GPa) crosslinked PILP-LCC scaffolds were similar to the mean value that was obtained for native bovine bone (5.5 ± 5.6 GPa). Future work is aimed to scale up production of PILP-LCC and test the biomaterial in a resorptive environment to determine its potential for use as a scaffold in bone regenerative engineering.

## Figures and Tables

**Table 1:** Liquid crystalline collagen (LCC) crosslink density as a function of *N*,*N*-(3-dimethylaminopropyl)-*N*′-ethyl-carbodiimide hydrochloride (EDC) concentration (n = 1 due to limited sample size).

LCC ID	EDC Conc. (mM)	EDC/NH_2_ collagen (mol/mol)	EDC/NHS (mol/mol)	Absorption (~340 nm)	Crosslink Density (%)
Blank	N/A	N/A	N/A	1.91	N/A
X0	0	0	0	2.81	0
X32	10	50	2	2.51	32
X51	50	50	2	2.35	51
X76	100	50	2	2.13	76

## Supplementary Materials

The following are available online at http://www.mdpi.com/1996-1944/11/7/1257/s1, Figure S1: Electron microscopy and optical images LCC long-range order.
