# Supplementary material for: Hierarchical Characterization and Nanomechanical Assessment of Biomimetic Scaffolds Mimicking Lamellar Bone via Atomic Force Microscopy Cantilever-Based Nanoindentation
Source: Materials (Basel). 2018 Jul 22;11(7):1257. doi: 10.3390/ma11071257 (PMC6073810; doi:10.3390/ma11071257)
Supplement: Supplementary file 1 [file materials-11-01257-s001.pdf]

Supplementary Materials

Hierarchical Characterization and Nanomechanical Assessment of Biomimetic Scaffolds Mimicking Lamellar Bone via Atomic Force Microscopy Cantilever-Based Nanoindentation

Brian Wingender [1], Yongliang Ni [2], Yifan Zhang [2], Curtis Taylor [2], and Laurie Gower [3,*]

[1] Department of Biomedical Engineering, University of Connecticut Health Center, Farmington, CT 06030-165, USA; wingender@uchc.edu
[2] Department of Mechanical and Aerospace Engineering, University of Florida, Gainesville, FL 32611, USA; niutouchaoren@ufl.edu (Y.N.); yifanzhang97@ufl.edu (Y.Z.); curtis.taylor@ufl.edu (C.T.)
[3] Department of Materials Science and Engineering, University of Florida, Gainesville, FL 32611, USA
* Correspondence: LGower@mse.ufl.edu; Tel.: +1-352-846-3336

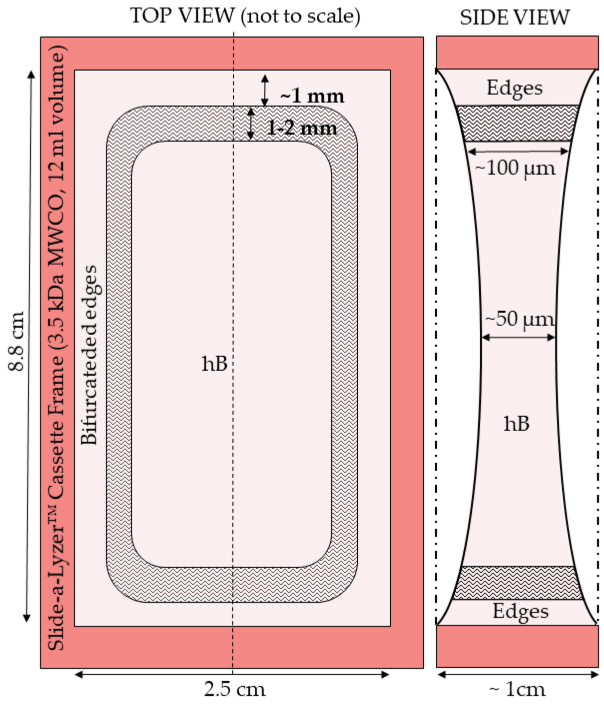

(**a**)

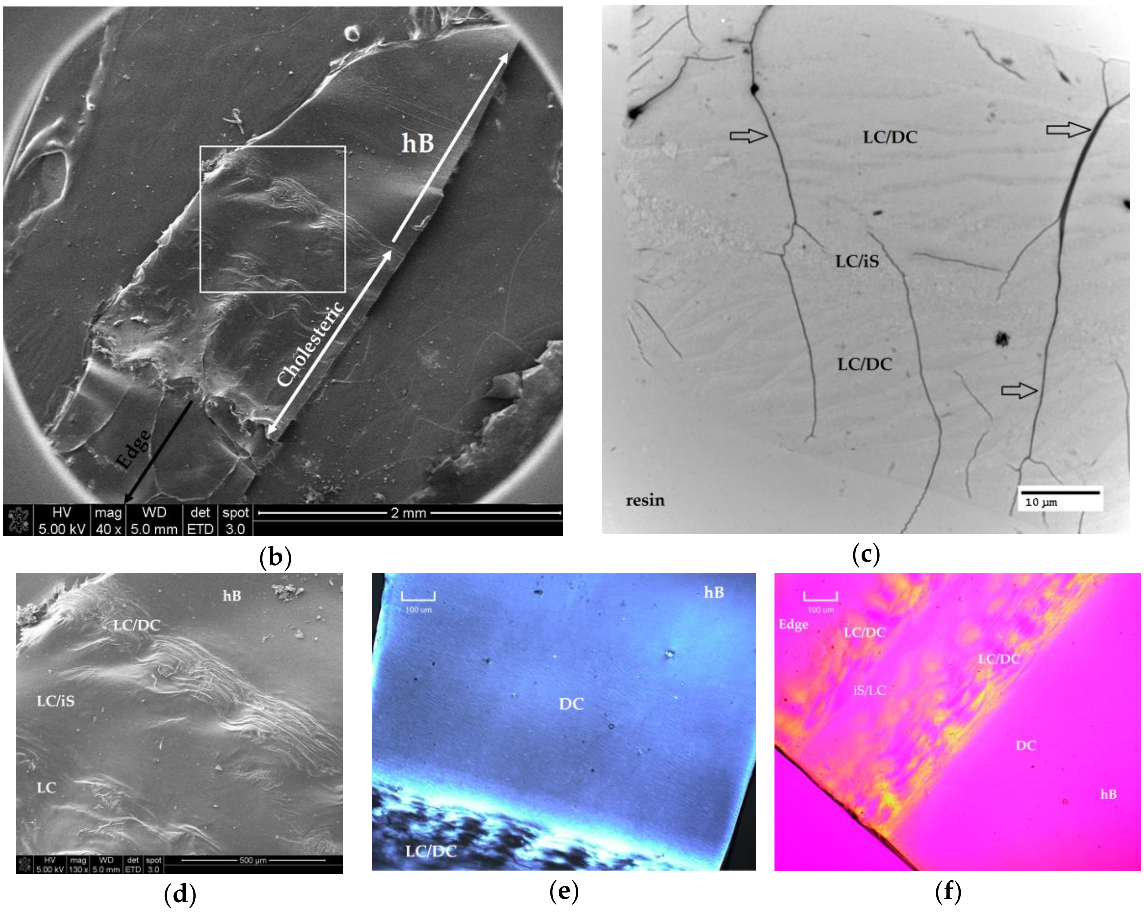

Figure S1. Electron microscopy and optical images of LCC long-range order. The current process for the generation of LCC films in Slide-A-Lyzer™ Dialysis Cassette results in inhomogeneous regions of cholesteric order, likely due to geometry restrictions imposed on the membrane by the rigid plastic frame. Mosser et al. conducted an in vitro fibrillogenesis study to determine how collagen concentration effected cholesteric organization in confined space [1]; their descriptive terminology will be used to help explain the cholesteric textures observed in this experiment. (**a**) Schematic illustration of dialysis cassette. Top view corresponds to the orientation shown for the images presented in (**b**), (**d**), (**e**), and (**f**) of this figure. The side view illustrates the change in membrane geometry after PEG dialysis, which causes the membrane surface to bow inward (solid lines) compared to its original position (straight dashed lines). At collagen concentrations of approximately 50–400 mg/mL, a well-defined cholesteric region roughly 1–2 mm in width (gray wavy pattern, top view) forms near the LCC film edges. A highly birefringent area (hB) comprises the central portion of the LCC film and is indicative of collagen concentrations exceeding 400 mg/mL. Bifurcated edges form near the LCC periphery as collagen is concentrated against both inner surfaces of the dialysis membrane. (**b**) Low magnification SEM top surface view of a specimen strip cut from the bulk of non-crosslinked LCC (X0); the cholesteric regions, delineated by the white double-sided arrow, appear as wavy striations and always form near the edges of the film. Single-sided arrows depict the location of the edges (black arrow) and central hB region (white arrow) with respect to the cholesteric ROI. (**c**) Low mag TEM image of LCC (X51) in cross-section, cholesteric regions appear as light and dark striations (LC/DC) while in the scaffold center a less organized region can be seen (LC/iS). Despite heavy metal staining, wrinkles in the section (dark lines, open arrows) decrease the image contrast making the long-range difficult to visualize in TEM. (**d**) Higher magnification SEM image of cholesteric regions marked by the white box in (**b**). (**e**) PLM image of cholesteric region of another specimen from the same sample showing typical birefringence patterns for dense cholesteric collagen reconstituted in vitro. (**f**) The same sample in (**d**) rotated by about 90 degrees clockwise, and imaged with a -plate inserted between the crossed-polars to show that the birefringence patterns are caused

by the shifting orientation of collagen. Definitions: LC—loose cholesteric; DC—dense cholesteric; hB—homogeneous birefringent area; iS – interconnected spherulitic.

References

1. Mosser, G.; Anglo, A.; Helary, C.; Bouligand, Y.; Giraud-Guille, M.M. Dense tissue-like collagen matrices formed in cell-free conditions. Matrix Biol. **2006**, *25*, 3—13.
